# Towards a sub-regional network of neuro-oncology in West Africa: An imperative to bridge the inequalities in care and research and to improve clinical outcomes

**DOI:** 10.1093/noajnl/vdag018

**Published:** 2026-01-27

**Authors:** Mèhomè Wilfried Dossou

**Affiliations:** Neurosurgery Department, Yopougon Training Hospital Center, Abidjan, Ivory Coast; Training and Research Unit in Medical Sciences of Abidjan, Felix Houphouët Boigny University, Ivory Coast; Neurosurgery Department, Martinique Training Hospital Center, Pierre Zobda Quitman Hospital, Martinique, France; Research Department of Sub-Saharan Africa Futures Neurosurgeons Association (SAFNA), Cotonou, Benin Republic

**Keywords:** Africa, global neurosurgery, governance, neuro-oncology, research

Dear Editor,

Brain tumors pose a major challenge in low- and middle-income countries (LMICs) due to limited resources and high costs, resulting in hampered service delivery of adequate neuro-oncologic care.^1^ Neuro-oncologic care requires a comprehensive, multidisciplinary team (MDT), including neurosurgeons, neuro-oncologists, radiation oncologists, neurologists, radiologists, pathologists, oncology nurses, and rehabilitation specialists. In cases where curative treatment options are limited, palliative care becomes essential, playing a central role in optimizing quality of life, psychological well-being, and end-of-life care. Timely access to palliative care has been shown to improve health-related quality of life and overall patient outcomes.^2^ Ensuring equitable access to neuro-oncologic care remains a major global health challenge, particularly in low- and middle-income countries, where limited resources, fragmented health systems, and delayed access to diagnosis and treatment result in poorer outcomes.^2^

Sub-Saharan Africa (SSA) faces major challenges in the management of central nervous system tumors due to limited diagnostic and therapeutic infrastructure, shortages of trained specialists, and weak health information systems. As a result, reported cases likely underestimate the true disease burden.^2^ In West Africa, these gaps are further exacerbated by structural, economic, and sociocultural barriers that widen disparities in access to care.^3^

To better characterize these gaps, we conducted a preliminary multicenter survey among neuro-oncology professionals health care from nine West African countries, namely Benin, Burkina Faso, Côte d’Ivoire, Ghana, Mali, Niger, Nigeria, Senegal, and Togo. The survey, presented at the Society for Neuro-Oncology Sub-Saharan Africa (SNOSSA) Congress held in Yamoussoukro, Côte d’Ivoire,^4^ included 35 participants comprising neurosurgeons, neurologists, medical oncologists, radiologists, and pathologists. The findings validate previously observed gaps (Figure 1). They are: A critically low neurosurgeon density (<1 per 1 000 000 inhabitants; up to 1 per 700 000 in Senegal), a limited access to advanced surgical techniques (awake surgery, stereotactic biopsy), and essential diagnostics such as immunohistochemistry (51%) and molecular biology (14%). It also appears that prolonged delays, often exceeding one month, for histopathology and molecular analyses and MDT meetings were available in only 34% of centers (Figure 2), despite strong demand (95%) for collaborative platforms and structured inter-university programs.

**Figure 1.** West Africa neuro-oncology indicators from our survey.

**Figure 2.**
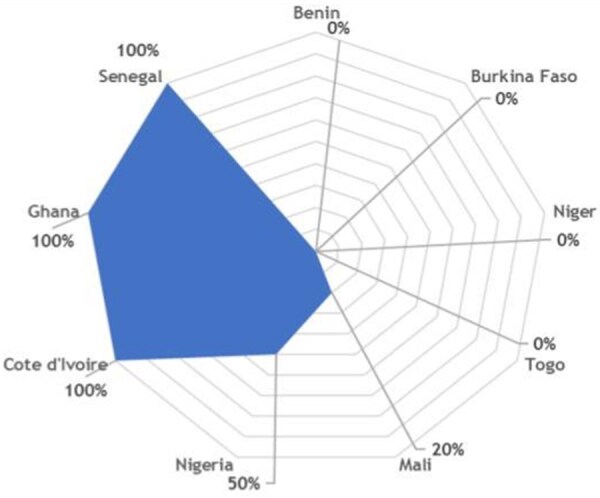
Distribution of the existence of a multidisciplinary consultation framework in neuro-oncology in the ECOWAS region.

These disparities are amplified by systemic constraints, including limited health budgets, fragmented referral pathways, lack of universal health coverage, and sociocultural factors that delay diagnosis and treatment. The resulting economic burden is substantial, as families often bear catastrophic out-of-pocket costs or resort to costly referrals abroad, while productivity losses impact national economies (Figure 3).

**Figure 3.** Factors associated with the lack of a multidisciplinary consultation framework in neuro-oncology in the ECOWAS region.

To address these challenges, we advocate for the establishment of a coordinated subregional neuro-oncology network in West Africa, built around three pillars. This network could be coordinated at a sub-regional level under the auspices of existing regional health bodies, such as the West African Health Organization (WAHO), in close collaboration with national referral centers.

First, the implementation of hybrid MDT (in-person and virtual) to optimize and support collaborative case discussions and second opinions.

Similar initiatives in the pediatric field have demonstrated their feasibility and impact. For example, an international tumor board via Zoom, launched in January 2021 between Washington University (St. Louis) and nine international sites (expanded to 20 institutions), allowed the review of 35 cases and the participation of 320 multidisciplinary experts.^5^ A recent review highlights the benefits of hybrid meetings (online and in person), facilitating real-time access to high-level expertise, especially in LMICs. In 2025, a multi-site survey (progress in neuro-oncological pediatrics) highlights the importance of MDT and communication between specialties in strengthening practices in the LMIC context.^6^ Based on these models, MDT meetings adapted to local resources could be implemented. These platforms would facilitate collaborative case discussions, second opinions, and shared decision-making while remaining sustainable and accessible. The use of telemedicine, mobile health technologies, simultaneous translation tools, and rotating bilingual moderators could help overcome geographic and linguistic barriers between Anglophone and Francophone countries.

Second, shared training programs to increase local skills and harmonize clinical practices.

Twinning programs between LMICs centers and high-income countries have demonstrated their benefits in improving the diagnosis and management of pediatric brain tumors.^7^ The global neurosurgery literature consistently highlights a critical workforce deficit in LMICs, far below the recommended ratio of one neurosurgeon per 200 000 inhabitants.^8^ In SSA, shortages of trained specialists, uneven workforce distribution, and limited access to subspecialty training contribute to delayed diagnosis and inadequate care.^2^ Sustainable solutions require long-term policy reforms combined with short-term strategies, including twinning programs such as regional fellowships, joint curricula, and standardized training programs developed in collaboration with regional accrediting bodies such as the West African College of Surgeons (WACS) and African and Malagasy Council for Higher Education (CAMES).

Third, resources sharing and setting up a regional tumor registry, promoting collaborative research and reducing inequalities.

Although neuro-oncology data are still poorly documented in sub-Saharan Africa, emerging technological innovations offer promising opportunities to offer new perspectives for diagnostic and health care management.^9^ In addition to government support, capacity-building and training initiatives are pivotal in enhancing healthcare access. These programs have seen significant growth in the region, strongly emphasizing forging enduring partnerships between countries and West African Health Organization (WAHO). These initiatives facilitate the exchange of expertise and training, contributing to the development of local healthcare expertise such as Yemaachi Biotech (Ghana) for molecular oncology, West African Center for Cell Biology of Infectious (University of Ghana) for genomic and biomarker research. The success of this sub-regional collaboration will constitute the central pillar for the establishment of the sub-regional Neuro-oncology registry and the harmonization care protocols. Such a registry could be developed progressively, starting with sentinel centers, and later expanded regionally to support epidemiological surveillance, quality improvement initiatives, and collaborative research.

On a global scale, the Global Task Force on Expanded Access to Cancer Care and Control in Developing Countries (GTF CCC), initiated by institutions from Harvard and the Fred Hutchinson Cancer Research Center, develops models for the expansion of oncological care in several countries including Rwanda, Malawi, Haiti, Mexico, or Jordan.^10^ These successful experiences and initiatives in LMICS-like settings need to be popularized and promoted in West Africa to reduce inequalities, standardize care and improve outcomes for patients with CNS tumors.

We therefore call on the global neurosurgery and neuro-oncology communities, as well as regional institutions such as WAHO, to support these initiatives through technical, academic, and financial partnerships. By leveraging hybrid MDTs, twinning programs, shared training, and collaborative data platforms, West Africa can build sustainable neuro-oncology capacity, standardize care pathways, and ultimately improve outcomes for patients with CNS tumors.

## Data Availability

All data will be made available upon request.
